# The influence of climate change on the future distribution of two *Thymus* species in Iran: MaxEnt model-based prediction

**DOI:** 10.1186/s12870-024-04965-1

**Published:** 2024-04-11

**Authors:** Naser Hosseini, Mansour Ghorbanpour, Hossein Mostafavi

**Affiliations:** 1 Department of Medicinal Plants, Faculty of Agriculture and Natural Resources, Arak University, Arak, 38156-8-8349 Iran; 2 Department of Biodiversity and Ecosystem Management, Environmental Sciences Research Institute, Shahid Beheshti University, Tehran, Iran

**Keywords:** Climate change, Future distribution, Species distribution models, *Thymus* genus

## Abstract

Within a few decades, the species habitat was reshaped at an alarming rate followed by climate change, leading to mass extinction, especially for sensitive species. Species distribution models (SDMs), which estimate both present and future species distribution, have been extensively developed to investigate the impacts of climate change on species distribution and assess habitat suitability. In the West Asia essential oils of *T. daenensis* and *T. kotschyanus* include high amounts of thymol and carvacrol and are commonly used as herbal tea, spice, flavoring agents and medicinal plants. Therefore, this study aimed to model these *Thymus* species in Iran using the MaxEnt model under two representative concentration pathways (RCP 4.5 and RCP 8.5) for the years 2050 and 2070. The findings revealed that the mean temperature of the warmest quarter (bio10) was the most significant variable affecting the distribution of *T. daenensis*. In the case of *T. kotschyanus*, slope percentage was the primary influencing factor. The MaxEnt modeling also demonstrated excellent performance, as indicated by all the Area Under the Curve (AUC) values exceeding 0.9. Moreover, based on the projections, the two mentioned species are expected to undergo negative area changes in the coming years. These results can serve as a valuable achievement for developing adaptive management strategies aimed at enhancing protection and sustainable utilization in the context of global climate change.

## Introduction

Within a few decades, the species habitat was reshaped at an alarming rate by climate change, leading to mass extinction, especially for sensitive species [1]. Biodiversity, agricultural production, and food security are predicted to alter expressively in response to a changing future climate globally [2, 3]. For example, high-diverse ecosystems in Melanesia Islands, which have the most diversified terrestrial ecosystems on the planet and hold more than half of the world’s coral species, have been vulnerable to climate change, habitat degradation, fragmentation, and loss over the last 50 years [4]. De Frenne et al. [5] revealed how forest microclimates will be influenced by climate change by exploring the interactions with land use changes across different biomes. Their results indicate the importance of global warming to ecological functions in local habitats, and biodiversity and ecosystem services cannot be ignored. Forecasting the impact of climate change on plant communities is an important area of study because it plays a crucial role in alerting scientists to make informed decisions in the face of future crises [6, 7]. Many new methods have been used to explore the species distribution pattern on the basis of global warming scenarios. Understanding how species will respond to climate change, including their distribution under future climate change scenarios, is vital for the effective management and conservation of biodiversity [8]. Species distribution models (SDMs) have been used extensively, with remarkable success, in understanding the influence of climate change on potential distribution of species [9, 10]. Among SDMs, the maximum entropy model (MaxEnt), which has shown superior performance compared to other models when dealing with limited sample sizes and presence-only data [11–13], has been widely used to assess ecological requirements, environmental responses, and habitat suitability of species. Iran with about 1.65 million square kilometer area, located on the Iranian Plateau, is a large country and after Turkey is the richest country of plant diversity in the Middle East [14]. Traditional medicine has always been very important in the Iranian culture and traditions, documented by many historical books describing Iranian traditional medicine as one of the oldest and richest alternative medicines [15]. Several valuable medicinal plants such as *Thymus* spp. distributed in Iran’s meadows [16]. Because they contain high levels of thymol and carvacrol, *T. daenensis* Celak and *T. kotschyanus* Boiss. & Hohen are valuable species in the genus [17, 18]. The populations of these species have severely declined in recent years, probably as a result of climate changes as well as excessive harvesting for food and medical purposes. So, understanding how species will adapt to climate change (e.g., how they will be distributed in future climate change scenarios) is crucial for effective biodiversity management and conservation [10, 19]. While SDMs have been widely employed for various purposes in Iran [20–24], to the best of the authors’ knowledge, the majority of prior studies on mentioned species were carried out locally, and no research has investigated the impact of climate change on the distribution pattern of *T. daenensis* and *T. kotschyanus* in the scale of Iran [21, 25–31]. As a result, the current study was aimed at achieving the following goals for the aforementioned *Thymus* species in Iran: (1) Identifying the most important climatic and ecological variables influencing the distribution pattern of these species; (2) predicting the appropriate areas of the *Thymus* spp. distribution pattern in Iran based on current climatic/environmental variables; (3) estimating possibly appropriate areas and Thymus spp. change trends under different climate conditions in the 2050s and 2070s; (4) Achievement to the aims provide valuable information for decision-makers involved in the future conservation planning.

## Materials and methods

### Study area

The study area is located within Iran and spans a vast surface area of 1.65 million square kilometers between 44°-64° E latitude and 25°-40° N longitude. Iran, situated in the arid zone of Middle East, holds the distinction of being the second-largest nation in the region. Iran’s altitude ranges from − 27 m above sea level (m.a.s.l) in the Caspian Sea basin to 5,670 m.a.s.l in the Alborz Mountain range. The country experiences an average annual rainfall of around 250 mm, as reported by Jamshidi and Samani [32]. The soil, topography, elevation, and water availability all affect the vegetation’s character. Iran with more than 8,000 species of vascular plants is the subject of this study. Over 30% of these species are endemic of Iran [33]. The country has a tenth of its land covered in forests [34]. There are several broad-leaved evergreens and broad-leaved deciduous trees in the area including; *Carpinus betulus* L. *Juglanse regia* L., *Ulmus glabra* Hudson, *Tilia platyphyllosa* Scop, *Quercus castaneaefolia* C. A. Mey, and *Fagus orientalis* Lipsky and thorny shrubs are also abundant. Woodlands, together with *Ulmus minor* Miller., *Pyrus* spp., *Celtis australis* L., *Juglanse regia*, *Pistacia atlantica* Desf., *Astragalus* spp. *Ferula* spp. and *Amygdalus scoparia* Soach. cover the Zagros Mountains. This area is a hot spot for crop wild relative and habitats of some medicinal plants such as *Thymus* spp [35, 36]. The ravines are host to many different species of creepers as well as *Salix excelsa* S. G. Gmelin, *Populus nigra* L., and *Platanus orientalis* L. On the intermediate dry plateau, thin stands of *Juniperus excelsa* M.Bieb., *Prunus lycioides (*Spach) C.K.Schneid., *Berberis vulgaris* L., *Cotoneaster* spp., and wild fruit trees are found. In the steppes, thorny plants like *Astragalus* spp. and *Acantholimon* spp. make up the ground cover, while in the hills and arid plains, medium altitudes are habitat to species of *Artemisia* spp., or wormwood. Below 900 m.a.s.l., *Ziziphus spina-chirsti* (L.) Willd. *Accacia* spp. and scattered plants are abundant. The water-retaining desert dunes support dense colonies of vegetation—waters, whether surface or subsurface, flow through forests. *Vitis vinifera* L., *Tamarix* spp., *Phoenix dactylifera* L., *Myrtus communis*, *Nerium oleander* L., and *Salix* spp., are all supported by desert [37].

### Species occurrence data

**Fig. 1:**
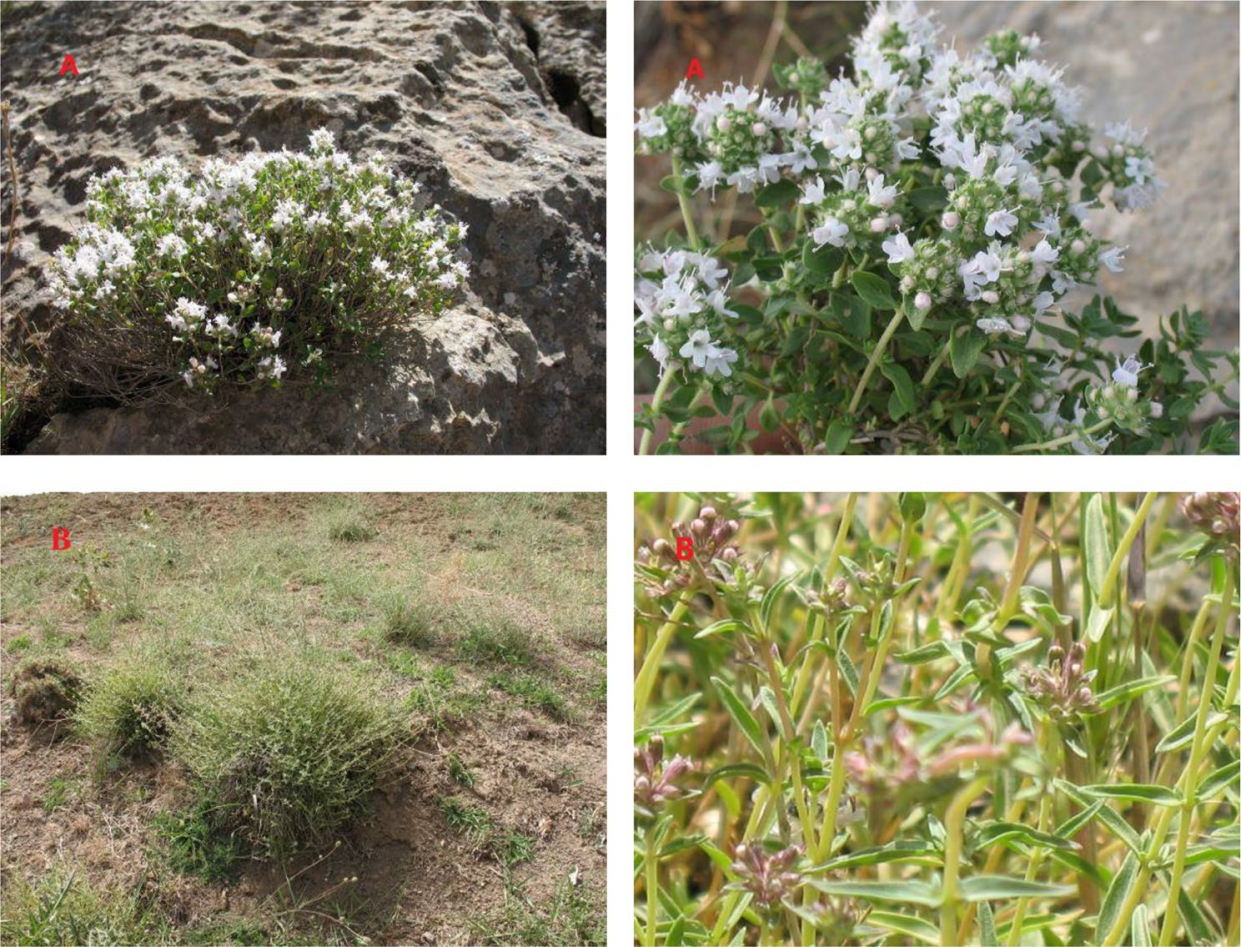
Photographs of *T. kotschyanus* (**A**) and *T. daenensis* (**B**) in the habitat

The genus *Thymus*, belonging to the *Lamiaceae* family and includes more than 215 species worldwide, is presented in Iranian flora by 18 species [38, 39]. *Thymus* species are mainly found in Iran’s western and northern highlands, including West Azerbaijan, East Azerbaijan, Ardabil, Zanjan, Kurdistan, Mazandaran, Golestan, Tehran, Hamadan, Markazi, and North Khorasan provinces [38, 39]. The carminative, digestive, antispasmodic, anti-inflammatory, emmenagogic, and tonic properties of thyme leaves and flowering parts are widely used in the folk medicine [40–42]. Essential oils are a group of remarkable substances found in *Thymus* spp. that have antifungal, phytotoxic, and insecticidal properties, encouraging their investigation and prospective usage in agricultural and food-related fields. *T. daenensis* Celak and *T. kotschyanus* Boiss. & Hohen, (Fig. 1) essential oil contains more than 60% of the valuable thymol and carvacrol compound [17, 18].

*T. daenensis* is a bushy perennial plant characterized by little hairs. It typically grows to a height of 15–30 cm and has opposite lanceolate to linear leaves with 18–20 mm long and 2–5 mm wide. Leaves have 2–3 pairs of dorsal vines. The inflorescence can be found at the end of the stem branches and consists of spherical or elongated clusters of reddish flowers. The calyx measures approximately 3–4.5 mm, tubiform or campanulate. The corolla is bilobed and measures 5–6 mm [38].

*T. kotschanus* is a perennial woody pulvinate plant with dense branches. It typically grows to a height of 6–12 cm and has opposite ovate leaves with 8–17 mm long and 4.5–7 mm wide. The inflorescence can be found at the end of the stem branches and consists of spherical clusters of white flowers. The calyx measures approximately 4.5–6 mm, tubiform or more and less campanulate. The corolla is bilobed and measures 6–7 mm [38].

**Fig. 2:**
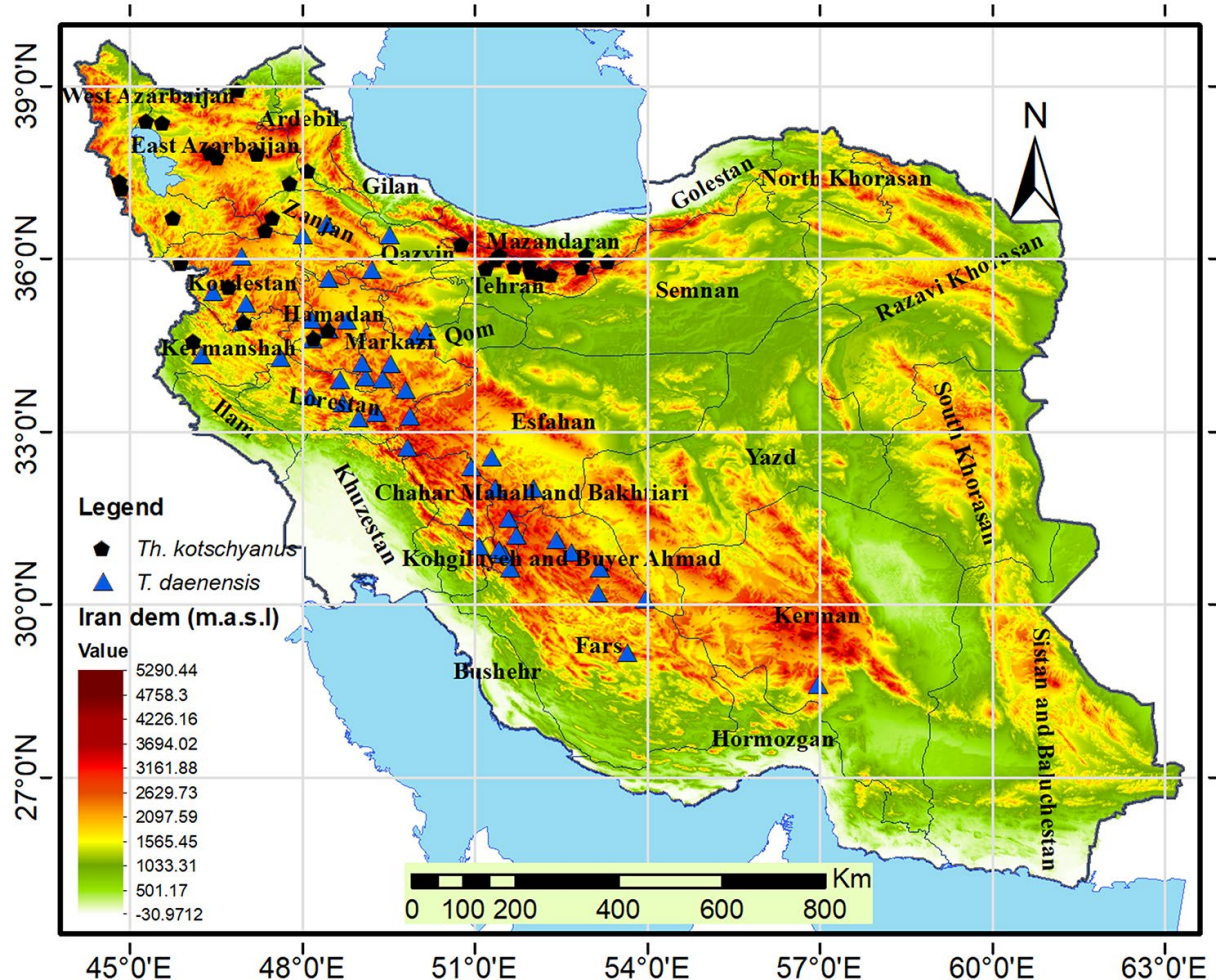
Distribution pattern of the two *Thymus* spp. in Iran (based on elevation map)

The presence points of the stated species were identified through an extensive range of sources, including direct field research, historical data available in the herbaria of HSBU, IRAN and TARI, (the herbarium acronyms follow the study of [43] and literature review [38, 39] [www.gbif.org]. It is essential to note that all points obtained from the literature and herbaria were confirmed through field verification. As reliable absence data for species distribution were not available, this study relied solely on presence data. After collecting the data occurrence, in order to avoid spatial autocorrelation among occurrence points, any points less than 1 km was removed initially and before modelling. For remove duplicate points we filtered occurrence data by randomly selecting a presence point within a single grid cell (i.e., 1 × 1 km) using ‘sdm’ package in R environment (Ver. 4.2.2). Finally, for *T*. *kotschyanus*, 32 occurrence points and for *T. daenensis*, 48 occurrence points were used to generate SDMs respectively (Fig. 2).

### Environmental variables

A total of 37 environmental variables including bioclimatic (www.worldclim.org), edaphic (www.soilgrid.org), and topographic variables (www.worldgrids.org) were initially selected at a spatial resolution of 30 arc-seconds (approximately 1 × 1 km). Afterwards, according to the ecology of species, available literature [17, 25, 29, 30, 32–35, 41, 42], expert judgment and correlation test, only five climatic factors, and one topographic factor were chosen to distribution modeling of the two *Thymus* species (Table 1). It is essential to be indicated that the co-linearity among variables was assessed using Pearson’s correlation coefficient (r), as suggested by previous researchers [6, 11, 44–47]. If two variables exhibited a high correlation (r>|0.70|), one of them was excluded to mitigate co-linearity [46].

**Table 1:** The most important environmental variables used in modeling

variables			
Code/Unit Bioclimatic variable		Code/Unit Topographic variable	
Bio1°C	Annual mean temperature	Aalt/m	Elevation
Bio2°C	Mean diurnal range (max. Temp – min. Temp)	*Slope/%*	**Slope**
Bio3°C	Isothermality (Bio2/Bio7)×100	Aspect (radian)	Aspect
Bio4°C	Temperature seasonality (SD × 100)	Solar/ kJ m-2 day-1	Solar radiation
Bio5°C	Max temperature of warmest month	**Code/Unit Edaphic variable**	
**Bio6°C**	*Min temperature of coldest month*	Depth /cm	Depth
Bio7°C	Temperature annual range (Bio5-Bio6) Bio7 (°C)	Depth.to.bedrock /cm	Depth to bedrock
Bio8°C	Mean temperature of wettest quarter	Cation.exchange.capacity /cmol/km	Cation exchange capacity
Bio9°C	Mean temperature of driest quarter Bio9 (°C)	Bulk density kg/dm3	Topsoil Bulk density
**Bio10°C**	*Mean temperature of warmest quarter*	Soil	Soil
**bio11°C**	*Mean temperature of coldest quarter*	Clay.content /%	Clay content
Bio12mm	Annual precipitation	Silt.content %	Silt content
bio13mm	Precipitation of wettest month	Sand.content %	Sand content
**Bio14mm**	*Precipitation of driest month*	Soil.organic.carbon.content %	Topsoil organic carbon content
Bio15mm	Precipitation seasonality	Coarse.fragments / cm^3^/dm^3^	Volumetric fraction of coarse fragments
Bio16mm	Precipitation of wettest quarter	Geology	Geology
Bio17mm	Precipitation of driest quarter	Land	Land
Bio18mm	Precipitation of warmest quarter	Land use	Land use
**Bio19mm**	*Precipitation of coldest quarter*	PH.index	PH.index

*Note* The *italicized* variables, were used in the two *Thymus* spp. modeling

### Distribution modeling

The MaxEnt model [48] was utilized for modeling the current and future habitat suitability of species. MaxEnt (Ver. 1.0–3) was utilized through the “dismo” package (Ver.1.3-9( (https://rspatial.org/raster/sdm/; Hijmans et al. [49] in the R programming environment (Ver. 4.2.2) [50]. The models were evaluated using 10-fold cross-validation to estimate errors and assess model consistency [51, 52]. In cross-validation, data were randomly divided into ten parts; nine parts were used for model fitting, and the fitted model was then used to evaluate the holdout part [53, 54]. The equal training sensitivity and specificity of MaxEnt output were considered as the proper threshold for the model’s prediction [55]. For predicting the current and future suitability, a single MaxEnt model with a full dataset was re-fitted. It is essential to be indicated that the process of modeling was repeated ten times with 10,000 background points in order to represent the variation in the environmental covariates and have stable model predictive performance [56].

To represent the future potential distributions of the *Thymus* spp., projected climate variables for 2050 (average for 2041–2060) and 2070 (average for 2061–2080) were utilized. The projections were derived from the average of 16 general circulation models (GCMs) under two greenhouse-gas emissions scenarios: semi-optimistic (RCP4.5) and pessimistic (RCP8.5). In fact, in order to reduce the uncertainty, the average of 16 general circulation models (GCMs) was applied. The environmental parameters were standardized to a common spatial resolution of 30 s of latitude/longitude. Suitability maps for each species under present and future climate scenarios were generated using ArcGIS software (Ver. 10.2).

### Model evaluation

To assess the accuracy of the modeling results, we computed the area under the curve (AUC) of the receiver operating characteristic (ROC) [57]. The AUC score is a powerful tool for measuring model performance because of its independence from threshold selection [58, 59]. The AUC shows the power of the model to discriminate presences from a random background [60]. The AUC ranges between 0 and 1.0, with 0.5 showing a random prediction performance and 1.0 indicating perfect discrimination. Values under 0.50 indicate models worse than random [46]. The percent contribution of the Jackknife test was used to assess the relative importance of each environmental variable influencing the models of specific species under current climate conditions [48], which is the best index for small sample sizes [61]. This analysis helps to understand the relative importance of different environmental factors in shaping the species’ habitats [62]. Additionally, the permutation importance (PI) was used to indicate the model’s dependence on each variable [48].

## Results

### The model evaluation

According to the AUC values, the modeling performance for the studied species was 0.927 for *T. daenensis* and 0.957 for *T. kotschyanus* according to training data (Figures not shown). The AUC showed that the performance of the model was excellent [63].

### Variables importance

**Fig. 3:**
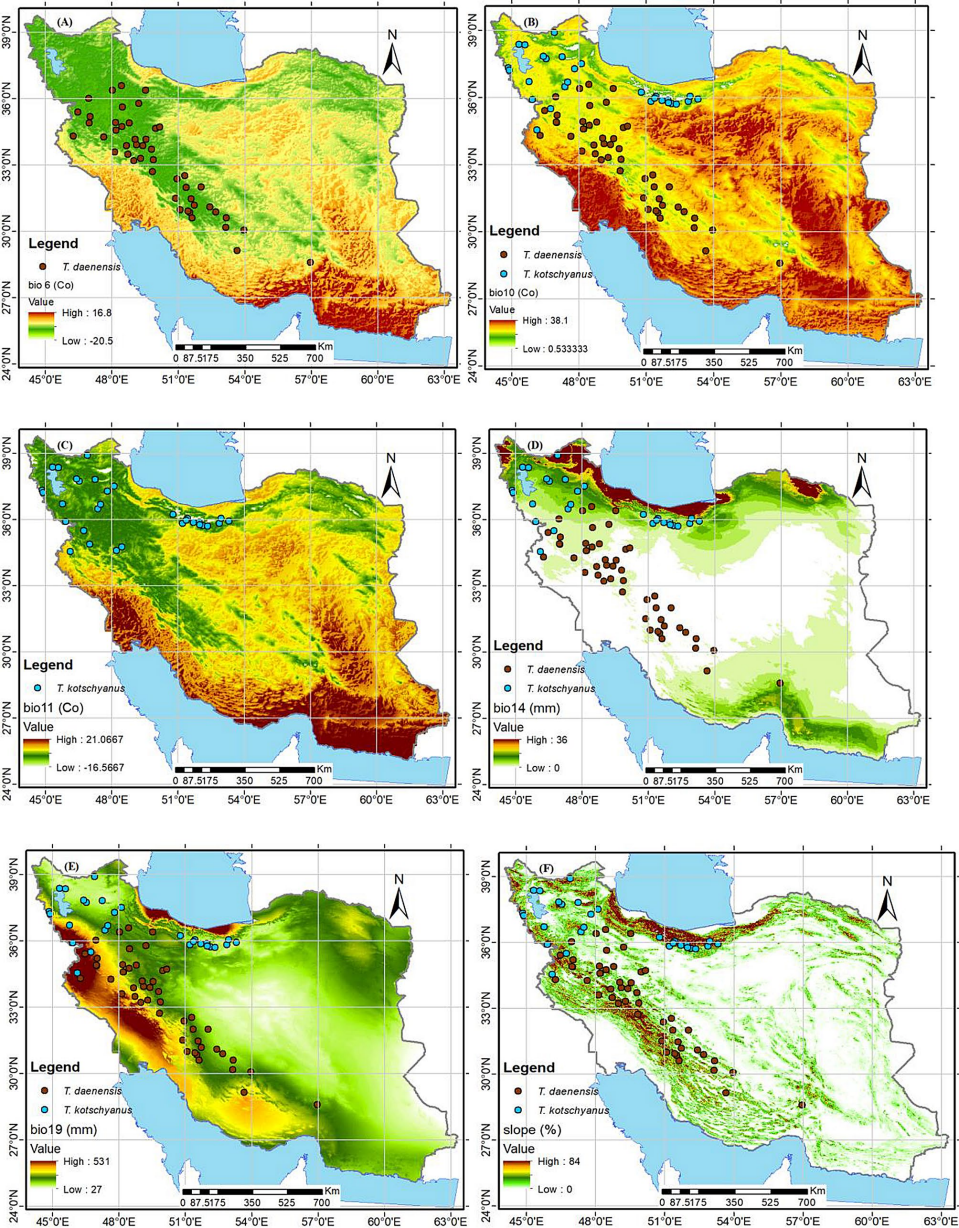
Environmental variables for modeling of the *Thymus* spp. Abbreviations are described in Table 1

Following the Pearson correlation test, each species was retained for modeling, with a total of six variables chosen for two species (Table 2). Given the present contribution (PC) values, the importance of the variables for each species varied remarkably (Table 2). The most important variables for *T. daenensis* were mean temperature of warmest quarter (bio10) (52.7%), the slope percentage (20.6%), and precipitation of driest month (bio14) (18.3%) (Fig. 3 panels B, D and F; Table 2). In addition, the model indicated that slope percentage (60.8%) and mean temperature of the coldest quarter (bio11) (28.5%) were the most restricting environmental variables based on the existing distribution pattern of *T. kotschyanus* (Fig. 3 panels F and C; Table 2;). According to Table 2; Fig. 3A and C, min temperature of the coldest month (bio6) was unique to *T. daenensis*, and mean temperature of the coldest quarter (bio11) was unique to *T. kotschyanus*, whereas the other four of the variables were shared by both species (Fig. 3).

**Table 2:** Percent contribution (PC) and permutation importance (PI) of the environmental variables for the *Thymus* spp

Environmental variable	Description	T. daenensis			T. kotschyanus	
		PC	PI		PC	PI
Bio6 ^o^C	Min temperature of coldest month	0.3		7.3	-	-
Bio10 ^o^C	Mean temperature of warmest quarter	52.7		67.6	3.4	2.4
Bio11 ^o^C	Mean temperature of coldest quarter	-		-	28.5	73.4
Bio14mm	Precipitation of driest month	18.3		15.7	6.1	1.4
Bio19mm	Precipitation of coldest quarter	8.1		3.3	1.2	0.8
Slope	Slope percentage	20.6		6.1	60.8	22.1

*Note* Dash (-) denote that some variables do not include for all species

### Predicted current potential distribution pattern

**Fig. 4:**
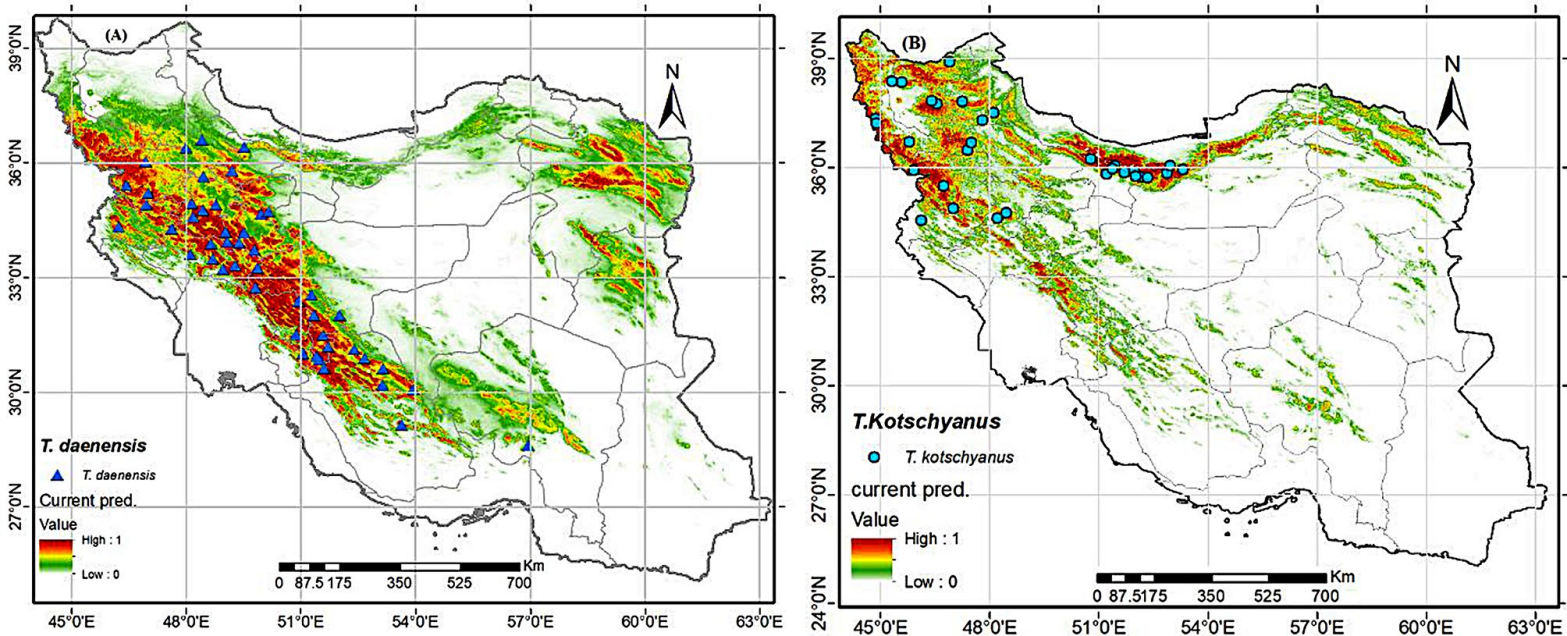
Map for potential current habitat suitability of the *Thymus* spp in Iran

MaxEnet modeling indicates that the current most suitable habitats for *T. daenensis* (Fig. 4A) are mountains of northwest and north of Iran (i.e., Qazvin, Zanjan, Kordestan, Kermanshah, Ilam, Hamadan, Markazi, Lorestan, Esfahan, Chaharmahal and Bakhtiari, Kohgiloyeh and Buyer Ahmad, Fars, Kerman, South Khorasan and Razvi Khorasan Provinces). The current most suitable habitats for *T. kotschyanus* (Fig. 4B) are mountains of west and northwest and north of Iran (i.e., West Azarbaijan, Eest Azarbaijan, Ardabil, Kordestan, Lorestan, Hamadan Tehran, Gilan, Mazandran, Golestan and North Khorasan Provinces). In summary, the current potential range of the two species exceeds their actual habitat by a substantial margin (Fig. 4).

### Predicted future potential distribution pattern

**Fig. 5:**
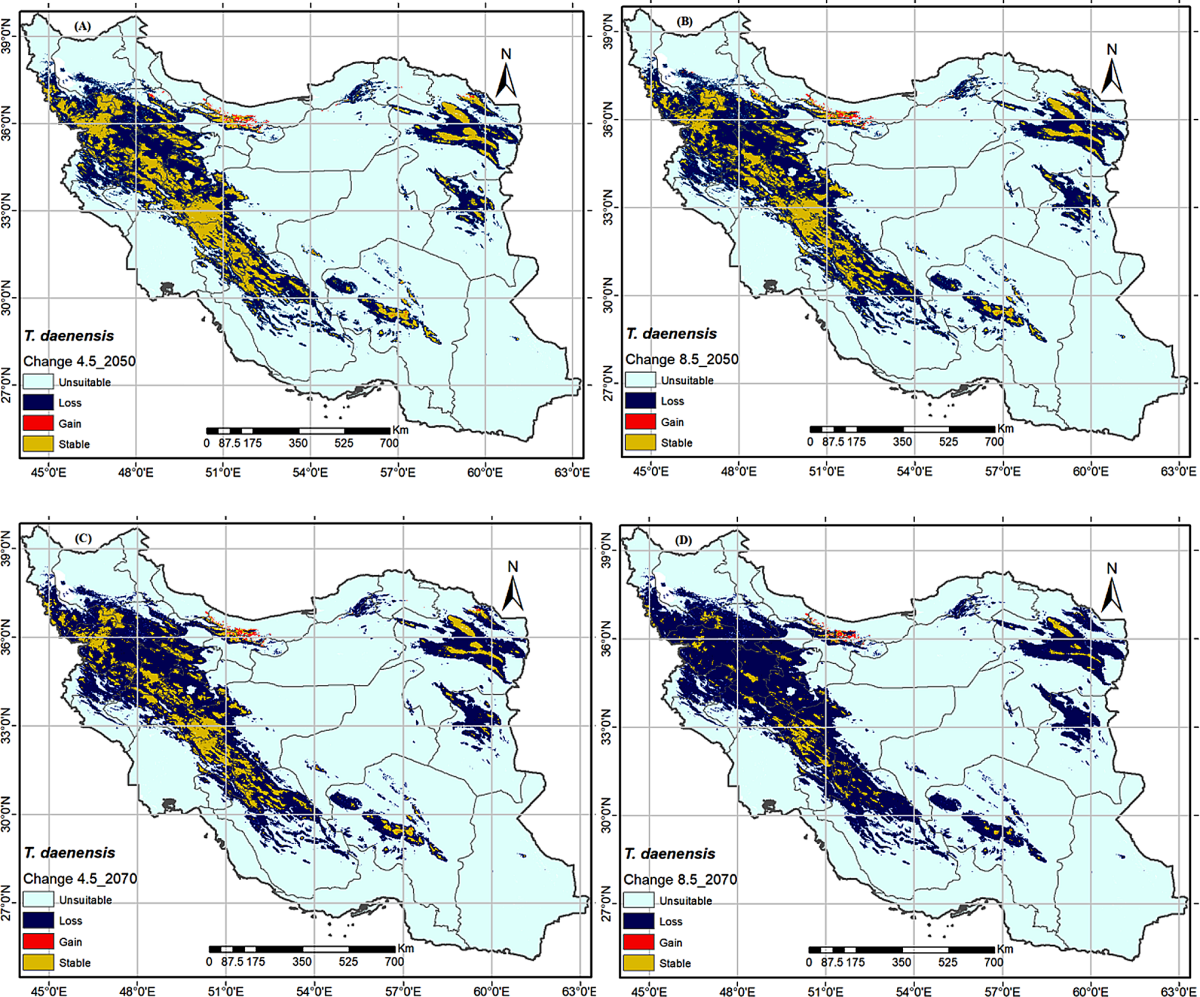
The future distribution map changes of *Thymus daenensis* in 2050 (average for 2041–2060) and 2070 (average for 2061–2080) under semi-optimistic (RCP4.5) and pessimistic (RCP8.5) scenarios

**Fig. 6:**
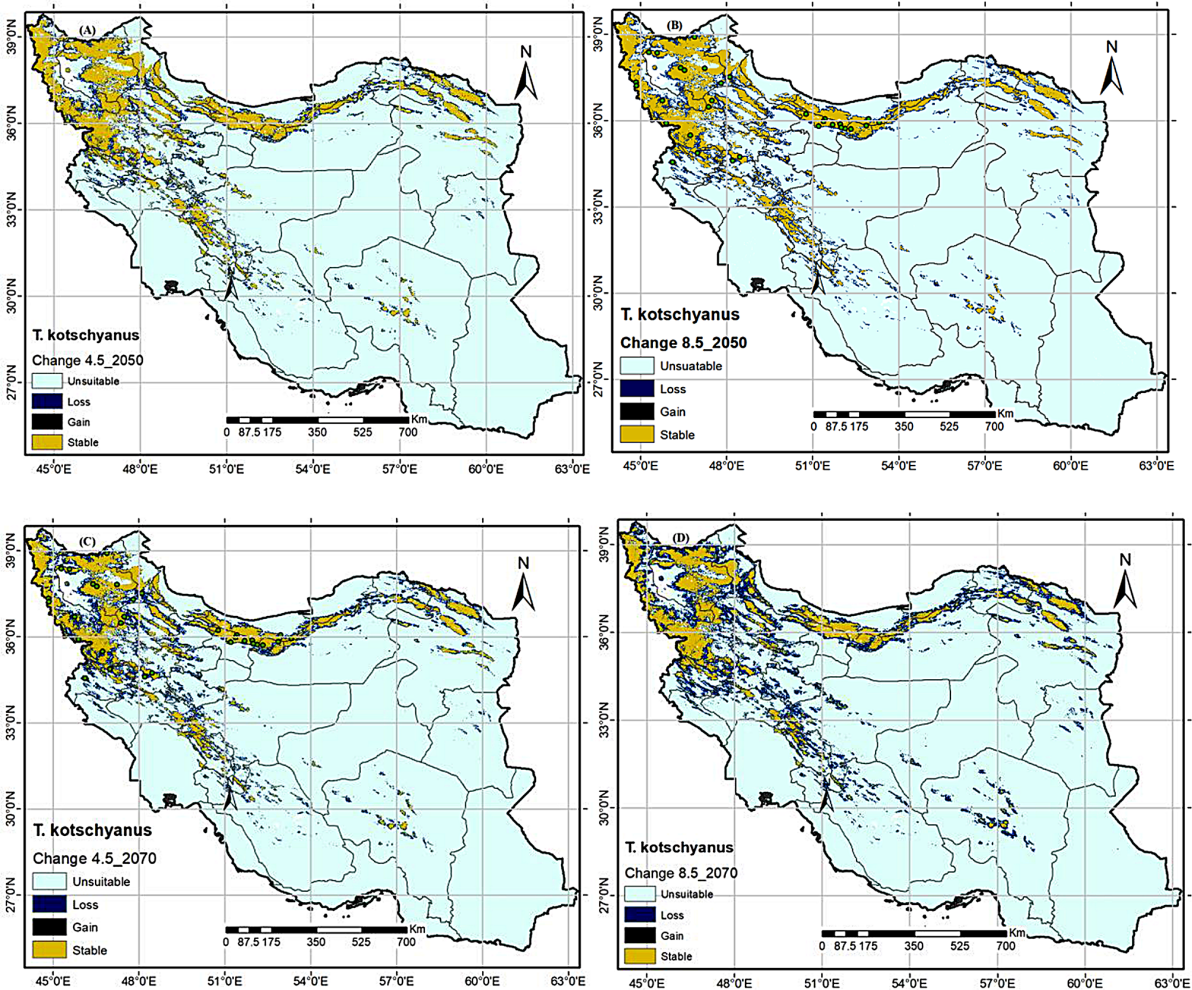
The future distribution map changes of *Thymus kotschyanus* in 2050 (average for 2041–2060) and 2070 (average for 2061–2080) under semi-optimistic (RCP4.5) and pessimistic (RCP8.5) scenarios

It is obvious that there is a gradual transition between scenarios and time ranges for each species (Figs. 5 and 6). Cwompared to the current potential habitat, tow *Thymus* spp. are projected to experience negative range changes in 2050 and 2070 under different RCP4.5 and RCP8.5 scenarios (Figures: 5 and 6), albeit with varying percentages (see Table 3). *T. daenensis* and *T. kotschyanus* have current distribution areas of 484,070 Km2 and 332,231 Km2, respectively. They will face the greatest losses in the RCP8.5 scenarios, reaching 41,682 Km2 and 142,805 Km2, or their distribution ranges decline 91.39% and 57.02%, respectively (Figs. 5 and 6; Table 3).

In compared to the current condition, provinces such as Qazvin, Kermanshah, Markazi, Kohgiloyeh and Buyer Ahmad, Fars, Kerman, and South Khorasan will be completely affected in RCP 8.5_2070, resulting the most adverse distribution range changes for *T. daenensis*, while this species’ distribution range will decline in lower altitudes of Zanjan, Kordestan, Lorestan, Hamadan, Esfahan, Chaharmahal and Bachtiari, and Razavi Khorasan provinces (Figs. 5 and 6). In contrast, new limited habitats in the highlands north of Tehran and south of Mazandaran provinces will be suitable for the spread of the aforementioned species. For *T. Kotschyanus* distribution range will decline in lower altitude parts of West Azarbaijan, East Azarbaijan, Ardabil, Kordestan, Lorestan, Hamadan Tehran, Gilan, Mazandran, Golestan, and North Khorasan provinces, and will be restricted to highlands of the mentioned areas (Fig. 6).

**Table 3:** The gain, loss, and range change of the two *Thymus* spp. under semi-optimistic (RCP4.5) and pessimistic (RCP8.5) climate change scenarios of 2050 (average for 2041–2060) and 2070 (average for 2061–2080)

Species	Scenario	Year	Current range (Km^2^)	Future range (Km^2^)	Loss		Gain		Range changes	
					Km^2^	%	Km^2^	%	Km^2^	%
*T. daenensis*	4.5	2050	484,070	167,726	319,162	65.93	2818	0.58	-316,344	-65.35
*T. daenensis*	8.5	2050	484,070	135,453	351,820	72.68	3203	0.66	-348,617	-72.02
*T. daenensis*	4.5	2070	484,070	114,698	371,864	76.82	2492	0.51	-369,372	-76.31
*T. daenensis*	8.5	2070	484,070	41,682	444,446	91.81	2058	0.43	-442,388	-91.39
*T. kotschyanus*	4.5	2050	332,231	215,812	116,426	35.04	7	0.0	-116,419	-35.04
*T. kotschyanus*	8.5	2050	332,231	213,389	118,870	35.78	28	0.01	-118,842	-35.77
*T. kotschyanus*	4.5	2070	332,231	180,953	151,283	45.54	5	0.01	-151,278	-45.53
*T. kotschyanus*	8.5	2070	332,231	142,805	189,428	57.02	2	0.0	-189,426	-57.02

*Note* Dash (-) refer to negative range change

## Discussion

### *Environmental variables affecting the distribution pattern of Thymus* spp

It is crucial to recognize the key environmental factors affecting the geographical distribution pattern of a species in terms of an ecological perspective [64, 65]. Based on the value of contribution, the projected findings revealed that the variables involved in suitable habitat for the two *Thymus* species were as follows. Slope percentage and mean temperature of the coldest quarter (bio11) were the dominant variables influencing distribution pattern of *T. Kotschyanus* (60.8% and 28.5%) respectively., while the mean temperature of the warmest quarter (bio10) (52.7%) and slope percentage (20.8%) were the dominant variables influencing dispersion pattern of *T. daenensis* (Table 3). According to Esfanjani et al. [66] the most important environmental factors affecting distribution pattern of *T. kotschyanus* were pH, elevation, precipitation and temperature variation. Consistent with our findings tow studied species are distributed in the slopes of the mountainous in the heights of the Iranian and Turanian regions (Fig. 3F) and it is very important that the soils are well drained [40]. In mountainous areas like Iran slope percentage as a key topographic factor is vital in regulating the spatial arrangement of soil nutrient, soil stability [67, 68], water [69, 70], heat, and sunlight, creating diverse microclimates with unique soil properties [11, 71–73]. According to studies conducted in the West and North-West of Iran *T. kotschyanus* grows in spots with sandy-loam and sandy clay-loam soils, with annual precipitation ranging from 161 to 832 mm, mean temperatures ranging from 10.5 to 19.1^o^C, and elevation varying from 1000 up to 3000 m.a.s.l [25, 26, 29, 74]. Consistent with our findings, temperature was the most important factor influencing the presence of *Thymus* species, followed by elevation and precipitation (for *T. kotschyanus*, precipitation was more important than elevation) [75]. According to Larti et al. [25], *T. kotschyanus* can grow in a wide range of elevation, PH, slope, and soil texture, particularly in sandy and rocky mountains. This species requires less annual precipitation than *T. daenensis* and is able to grow in arid mountains, but it requires a cold winter to grow (Fig. 3C). Corticchiato et al. [76] stated that the main factors affecting the distribution pattern of *Thymus* species involve climate, altitude, soil type, soil texture, organic matter and calcium content of soil in east regions of Spain. Boira & Blanquer [77] pointed out that some factors involving elevation, soil texture, and climate affect development of *Thymus piperella* in Spain.

Our finding indicated, mean temperature of the warmest quarter (bio10) (52.7%), slope percentage (20.8%) and precipitation of the driest month (18.3%) were the dominant variable influencing the distribution pattern of *T. daenensis* (see Table 3). According to Arvin et al. [78] research, this species occurs in alpine regions at altitudes ranging from 1000 to 2400 m.a.s.l., with an average annual temperature of 11.9–17.7 ^o^C, an annual minimum temperature of 4.4–10.7 ^o^C, and precipitation ranging from 200 to 700 mm/year. Altitude, slope percentage and minimum mean temperature are important ecological variables in the mentioned and other *Thymus* species dispersion pattern [26, 27, 78–80].

### Predicting the current distribution potential of Thymus spp

The MaxEnt model has been widely used in ecology, conservation biology, evolutionary biology, and invasive species management [48]. Previous studies reported that the MaxEnt model was commonly implemented to predict potential distributions in many plant species in several national-scale studies, from Iran [36], Tunisia [81], China [82] and east Asia [83]. *Thymus* spp. are widely regarded as one of the most important spices and food preservatives in the food industry. Plant characteristics such as essential oil content and yield, and physiological and morphological traits may be affected by a variety of factors including climatic, soil conditions and genetics [75, 80, 84, 85]. These characteristics changes clearly depend on mean temperature and temperature extremes in plant’s ecosystem [80].

The MaxEnt model results identified that the current highly suitable habitats for *T. daenensis* were mostly in the west, center and the northeast of the country but, the actual presence range of the species does not include the northeast. These regions are characterized by elevation 1000–2500 m.a.s.l, precipitation of driest month (bio14) < 10 mm, mean temperature of the warmest quarter between 15 and 28 C^o^ and slope percentage > 3 < 23% (see Fig. 3). Nadjafi et al. [86] found that the optimal temperature for *T. daenensis* seeds germination was 20 °C. Temperatures above the optimum (22 °C) appear to be the outcome of secondary dormancy in the seeds of the mentioned species. Consistent with our findings according to Majd et al. [30] research, the best-growing conditions for the aforementioned species are foothills with average minimum and maximum temperatures of 12 and 23 degrees Celsius, followed by mountain areas with average minimum and maximum temperatures of 9 and 24 ^o^C.

Based on modeling suitable habitats for *T. kotschyanus* are in the west, north, and northeast of the country. However, the actual presence range of the species does not include the northeast (Fig. 3). These regions characterized by slope percentage > 5%, altidude > 1500 m.a.s.l and mean temperature of the coldest quarter < 5 C^o^ (Fig. 6). According to Fig. 4B, regions with high summer rainfall are unsuitable for this species’ growth, so in the north, regions like the Caspian Sea coast, which has a low elevation and high summer precipitation, are not suitable for this species’ dispersal. Consist whit our finding *T. kotschyanus* thrives in rocky slopes of the Irano-Turanian mountains, where it can reach elevations of up to 3000 m. These mountains have calcareous sandy loam soils and a cold, dry climate [25, 38].

### Prediction of the future potential distribution pattern of Thymus spp

In compared to the current condition, provinces such as Qazvin, Kermanshah, Markazi, Kohgiloyeh and Buyer Ahmad, Fars, Kerman, and South Khorasan will be completely affected in.

In the previous research, it has been observed that global climate change influences the future distribution pattern of plant species, with varied responses among different species [87]. The MaxEnt model revealed that the suitable habitats of the tow *Thymus* species reduce in 2050 and 2070 under semi-optimistic (RCP4.5) and pessimistic (RCP8.5) scenarios (Table 3). The distribution areas of *T. daenensis* and *T. kotschyanus* changed the most under the RCP 8.5–2070 scenario. According to RCP 8.5–2070, climate change will cause *T. daenensis* to lose approximately 91.39% of its current range, reducing it from 484,070 to 41,682 km^2^ (Table 3). While this species’ distribution range will decline in lower altitudes of Zanjan, Kordestan, Lorestan, Hamadan, Esfahan, Chaharmahal and Bachtiari, and Razavi Khorasan provinces (Fig. 5). In contrast, new limited habitats in the highlands north of Tehran and south of Mazandaran provinces will be suitable for the spread of the aforementioned species. Climate change, as projected by the RCP 8.5–2070 scenario, will reduce *T. kotschyanus’* distribution range by 57%, from 332,231 to 142,805 km^2^ (Table 3). *T. Kotschyanus* distribution range will decline in lower altitudes of West Azarbaijan, East Azarbaijan, Ardabil, Kordestan, Lorestan, Hamadan Tehran, Gilan, Mazandran, Golestan, and North Khorasan provinces, and will be restricted to highlands of the mentioned areas (Fig. 6).

*Thymus* species have a limited ecological niche and do not prefer marginal habitats [27]. A diverse range of mountainous plant communities have been reported around the world [88, 89]. A logical consequence is a shift in the spatial distribution of the species towards higher elevations to benefit from higher precipitation and cooler temperatures, resulting in more adapted plant development. Previous research has also detected an elevational shift in plant community distribution pattern under climate change scenarios in Iran and around the world, especially in mountain habitats [21, 88, 90]. The investigated *Thymus* species’ habitats are declining in the foothills, particularly at lower latitudes. *T. kotschyanus* is an alpine species found in sandy and rocky mountains at elevations ranging from 1500 to 3000 m above sea level. Temperature is the most important factor influenced the presence of *Thymus* species, followed by elevation and precipitation but for *T. kotschyanus*, the importance of precipitation is greater than elevation [75].

Highland environments remain suitable for alpine species, while for *T. daenensis* cool foothills with annual precipitation > 250 mm, annual mean temperature < 15 °C, annual minimum temperature < 9 °C, and elevation ranges between 1670 and 2000 m.a.s.l. are suitable areas [30, 78]. As a foothill’s species, climate change is having a significant impact on the distribution range of the species that are unable to migrate to the highlands. Germination behavior is an important part of a species’ regeneration strategy [91], and it is influenced by a variety of inherent and external factors [92, 93]. Temperature is the most vital of these elements, influencing the maximum germination percentage and rate of germination [94]. If climatic factors such as temperature and precipitation change in a region beyond the tolerance of a species’ plasticity, changes in species distribution may be unavoidable. It has been shown that plant species have shifted their altitudinal and latitudinal ranges in response to climate change [95], but there is also likely to be a rapid increase in extinction risk [96]. Increasing evidence indicates that the global average temperature is rising and precipitation is declining, in part due to increased greenhouse gas emissions [97]. Nadjafi et al. [86] found that 20 °C was the optimal temperature for *T. daenensis* seeds. It seems that increasing temperature over the optimum (22 °C) is the result of secondary dormancy in seeds of the species. Based on Sharifi Ashoorabadi et al. [98] studies highest germination percentage of thyme seeds occurs at temperatures ranging from − 4 to 4 degrees, and at temperatures ranging from − 8 to more than 4, the germination ratio decreases significantly. *Thymus* species are most common in cool to moderate annual temperatures in elevated area [75]. So, maybe climate change has a negative impact on thyme distribution range by reducing seed germination.

Plants with wider ecological niches such as *T. kotschyanus*, in particular, will be better able to adapt to climate change than species with limited ecological niches like *T. daenensis* [99, 100]. For instance, global warming has improved the habitat suitability of *Ruscus aculeatus* in Sardinia [101] and *Homonoia riparia* in China [58]. Future climate change was predicted to result in a significant reduction in suitable habitats for many other species, including *Fritillaria cirrhosa* in China [102], *Artemisia aucheri*, *A. sieberi*, and *Daphne mucronata* in Iran [99, 103]. This study employed two different climate change scenarios, RCP 4.5 and RCP 8.5, to evaluate the potential impacts of climate change on *Thymus* species in the future. RCP4.5 represents a relatively moderate and plausible scenario, considering greenhouse gas emissions mitigation, while RCP8.5 portrays a high-emission scenario. Considering the substantial differences between these two scenarios, the projected outcomes for the *Thymus* species are different. Under the RCP8.5 scenario, there is a higher projected loss of suitable areas for *T. daenensis* and *T. kotschyanus* compared to the RCP 4.5 scenario for both study periods, as indicated in Table 3. This suggests that the more severe greenhouse gas emissions scenario leads to severe adverse effects on the suitable habitats of these two *Thymus* species. However, range changes, rather than retractions, are the most important information to consider when effective conservation measures are planned [59, 104].

### Research limitations

We must recognize and acknowledge several limitations in our study. MaxEnt modeling has proven to be very effective at determining habitat use and species distributions for a variety of species and localities. Because it relies only on presence data, it lacks many of the complications associated with presence-absence analytical methods. MaxEnt modeling has frequently outperformed a number of other approaches that rely on presence-only data, it is relatively insensitive to spatial errors associated with location data, and it can produce useful models with as few as five locations. However, MaxEnt needs to develop methodology for selecting the best approximating model, and establish protocol for assessing habitat selection based on repeated sampling of individuals [105]. Another limitation of this research was that the chosen set of 6 environmental variables may not encompass all the factors influencing the geographic distribution pattern of *Thymus* spp. In other words, apart from the selected environmental variables, various other factors, such as species interactions [106], human activities such as over grazing, the habitat fragmentation and other land- use changes [107], pasture fire and distance from streams [11], may exert a critical influence on the distribution pattern of the *Thymus* spp. However, it is crucial to understand that the predicted suitable habitat area from the model may not always perfectly coincide with the actual habitat where the species is found [108, 109]. This discrepancy can arise due to uncertainties and internal assumptions within the species distribution models, highlighting the need for further research and consideration of these factors to improve the accuracy of such predictions.

## Conclusions

The leaves, flowers, and essential oils of *T. daenensis* and *T. kotschyanus* are widely used as herbal tea, spices, flavoring agents, and medicinal herbs. *Thymus* oils and extracts are also frequently utilized in the pharmaceutical, perfume, and cosmetic sectors, as well as for flavoring and protecting food products. Climate change has significant effects on the suitable habitats of *T. kotschyanus* and *T. daenensis*, causing these species to lose the majority of their distribution range in the future. More than half of the *Thymus* species are still obtained from wild land in the country, and they are frequently overharvested. The identification and analysis of appropriate habitats for the species production, along with promoting the cultivation of these valuable species in these habitats through support programs such as insurance, will have multiple benefits. Firstly, it will aid in the restoration of the species’ natural habitats by reducing the frequency of harvests. Secondly, it will foster an increase in the population of these species, thereby reducing the likelihood of their extinction. In this regard, it is suggested that large-scale cultivation of the two *Thymus* spp. in rangelands is required. The first step to expand cultivation of plants is the selection of new desirable areas. we can conclude that the best place for the production of quantity effective materials this plant in order to attain the best results, is the height between 1600 and 2400 m above sea level in north of Fars, Eshahan, Hamadan, Kordestan and North Khorasan provinces for *T. daenensis* and for *T. Kotschyanus*, West Azarbaijan, East Azarbaijan, Ardabil, Kordestan, Lorestan, Hamadan Tehran, Gilan, Mazandran, Golestan, and North Khorasan provinces. Moreover, by implementing protected areas in the mentioned provinces, it is possible to preserve the genetic diversity of the species and guarantee their survival.

## Data Availability

The raw data of this article will be made available by corresponding authors, according to the personal requests.

## Declarations

### Identification of the collected plant materials

The identification and authentication of thyme as a species was done according to Botanical Standards (Rechinger 1982) and vouchered in Arak University Herbarium (A1x).

## References

[1] Muluneh MG (2021). Impact of climate change on biodiversity and food security: a global perspective—a review article. Agric Food Secur.

[2] Vermeulen SJ, Campbell BM, Ingram JSI (2012). Climate change and food systems. Annu Rev Environ Resour.

[3] Wiebe K, Robinson S, Cattaneo A. Climate change, agriculture and food security: impacts and the potential for adaptation and mitigation. Sustainable food Agric, pp. 55–74, 2019.

[4] Bhattarai U. Impacts of climate change on biodiversity and ecosystem services: direction for future research. Hydro Nepal, pp. 41–8, 2017.

[5] De Frenne P (2021). Forest microclimates and climate change: importance, drivers and future research agenda. Glob Change Biol.

[6] Peng Z, Zhang Y, Zhu L (2023). Spatial and temporal patterns of the sensitivity of radial growth response by *Picea schrenkiana* to regional climate change in the Tianshan Mountains. J Res.

[7] Rahman W, Magos Brehm J, Maxted N (2023). The impact of climate change on the future distribution of priority crop wild relatives in Indonesia and implications for conservation planning. J Nat Conserv.

[8] Beridze B (2023). Biodiversity protection against anthropogenic climate change: conservation prioritization of Castanea sativa in the South Caucasus based on genetic and ecological metrics. Ecol Evol.

[9] Hama AA, Khwarahm NR (2023). Predictive mapping of two endemic oak tree species under climate change scenarios in a semiarid region: range overlap and implications for conservation. Ecol Inf.

[10] Naudiyal N (2021). Potential distribution of Abies, Picea, and Juniperus species in the sub-alpine forest of Minjiang headwater region under current and future climate scenarios and its implications on ecosystem services supply. Ecol Ind.

[11] Momeni Damaneh J, Ahmadi J, Rahmanian S, Sadeghi SMM, Nasiri V, Borz SA (2022). Prediction of wild pistachio ecological niche using machine learning models. Ecol Inf.

[12] Khan AM (2022). MaxEnt modelling and impact of climate change on habitat suitability variations of economically important Chilgoza Pine (Pinus gerardiana Wall.) In South Asia. Forests.

[13] Ahmadi M, Hemami M, Kaboli M, Shabani F (2023). MaxEnt brings comparable results when the input data are being completed; Model parameterization of four species distribution models. Ecol Evol.

[14] Jafari SM, Akhani H (2008). Plants of jahan nama protected area, Golestan Province, N. Iran. Pak J Bot.

[15] Ameri A, Heydarirad G, Mahdavi Jafari J, Ghobadi A, Rezaeizadeh H, Choopani R (2015). Medicinal plants contain mucilage used in traditional persian medicine (TPM). Pharm Biol.

[16] Nobakht SZ, Akaberi M, Mohammadpour AH, Moghadam AT, Emami SA (2022). Hypericum perforatum: traditional uses, clinical trials, and drug interactions. Iran J Basic Med Sci.

[17] Ghasemi G (2020). Composition, antifungal, phytotoxic, and insecticidal activities of thymus kotschyanus essential oil. Molecules.

[18] Bahreininejad B, Mirza M (2019). Effects of ecological factors on essential oil components of several genotypes of Thymus daenensis celak using ordination technique. Iran J Med Aromatic Plants Res.

[19] Shi X (2023). Prediction of the potentially suitable areas of Litsea cubeba in China based on future climate change using the optimized MaxEnt model. Ecol Ind.

[20] Asgarian A, Soffianian A (2023). Past and potential future distribution of white mangroves in an arid estuarine environment: integration of Maxent and CA-Markov models. Mar Policy.

[21] Behmanesh B, Tabasi E, Fakhireh A, Khalasi Ahvazi L (2019). Modeling the distribution of medicinal plant species of Thymus kotschyanus and Achillea millefolium using ENFA and Logistic Regression. J Plant Ecosyst Conserv.

[22] Bazrmanesh A, Tarkesh M, Bashari H, Poormanafi S (2019). Effect of climate change on the ecological niches of the climate of Bromus Tomentellus Boiss using Maxent in Isfahan province. J Range Watershed Managment.

[23] Mohammady M (2021). Modeling and prediction of Habitat Suitability for Ferula gummosa Medicinal Plant in a Mountainous Area. Nat Resour Res.

[24] Zeraatkar A, Khajoei Nasab F. Mapping the habitat suitability of endemic and sub-endemic almond species in Iran under current and future climate conditions. Environ Dev Sustain, pp. 1–18, 2023.

[25] Larti M, Ghasempour S, Sharifi Ashorabadi E, Alizadeh B (2013). The study of some ecological characteristics of Thymus kotschyanus Boiss. Et hohen and Thymus pubescens Boiss. & Kotschy ex Celak in West Azarbaijan. Iran J Med Aromatic Plants Res.

[26] Darvishi L, Zare Chahouki MA, Jafari M, Azarnivand H, Yousefi Valikchali M (2013). Study on the Environmental Factors Contributing to distribution of Thymus kotschyanus in Taleghan Basin, Iran. J Rangel Sci.

[27] Zare Chahouki MA, Abasi M (2016). Habitat suitability modeling for Thymus kotschyanus Boiss. & Hohen. Using ecological-niche factor analysis (case study: rangeland of middle Taleghan). Iran J Med Aromatic Plants Res.

[28] Nazari S, Jafarian Z, Alavi J, Naghi poor AA (2021). The impact of Climate Change on the Geographic distribution of *Thymus kotschyanus* (Boiss and Hohen) using ensemble modelling. Desert Manage.

[29] Yari R, Dashti M, Zeynabi M, Parsa Mohebi SM, Azizi N (2021). Autecology study of Thymus kotschyanus in Rangeland ecosystems of Boshrouyeh city, South Khorasan Province. Technol Med Aromatic Plants Iran.

[30] Majd M, Khoshhal Dastjerdi J, Sefidkon F, Lebasschi M, Baratian A (2021). Determination of the effects of climate on growth and phonological stage of Daenensis Thymuse for cultivation in different geographical regions. Phys Geogr Res Q.

[31] Jamshidi Z, Samani N (2022). Mapping the spatiotemporal diversity of precipitation in Iran using multiple statistical methods. Theoret Appl Climatol.

[32] Jamshidi Z, Samani N. Mapping the Spatiotemporal Diversity of Precipitation in Iran, 2021.

[33] Noroozi J, Moser D, Essl F (2016). Diversity, distribution, ecology and description rates of alpine endemic plant species from Iranian mountains. Alp Bot.

[34] Sharifi M. An Overview of Ecological Potential and the Outstanding Universal Value of Forests Resources of I.R. Iran with respect to Climate Change, *Forests, Rangelands and Climate Change in the Near East Region. Natural Parks and Protected Areas High Council Member of FRWO. Regional Workshop. 20–22 September 2011*, 2011.

[35] Sayadi S, Mehrabian A, Mostafavi H (2022). Diversity centers and distribution patterns of Eudicot crop wild relatives of Iran: priorities for conservation and important plant areas. J Wildl Biodivers.

[36] Hosseini N, Mehrabian A, Mostafavi H (2021). The distribution patterns and priorities for conservation of monocots Crop Wild relatives (CWRs) of Iran. J Wildl Biodivers.

[37] White F, Léonard J (1991). Phytogeographical links between Africa and Southwest Asia. Flora et vegetatio Mundi.

[38] Jamzad Z (2012). FLORA OF IRAN(lamiaceae).

[39] Jalas J. Thymus in KH Rechinger, editor, Fl. Iran. vol. 150: 532–551. Graz, 1982.

[40] Ghasemi Pirbalouti A, Emami Bistghani Z, Malekpoor F (2015). An overview on genus Thymus. J Med Herbs.

[41] Manukyan A (2019). Secondary metabolites and their antioxidant capacity of caucasian endemic thyme (Thymus transcaucasicus Ronn.) As affected by environmental stress. J Appl Res Med Aromatic Plants.

[42] Elahian F, Yazdinezhad A, Moein-Albokay Tusi N, Nouri Z, Mirzaei SA (2020). Variety of antibacterial and antifungal activity of Thymus kotschyanus essential oil collected from fourteen regions of Iran. J Birjand Univ Med Sci.

[43] Thiers B. Index Herbariorum: a global directory of public herbaria and associated staff. New York Garden’s Virtual Herbarium, *New York Garden‘s Virtual Herbarium* http://sweetgum.nybg.org/ih, 2022.

[44] Aminzadeh M, Amiri F, Abadi EA, Mahdevi K, Fadai S (2010). Factors affecting on essential chemical composition of Thymus kotschyanus in Iran. World Appl Sci J.

[45] Elith J (2006). Novel methods improve prediction of species’ distributions from occurrence data. Ecography.

[46] Elith J, Phillips SJ, Hastie T, Dudík M, Chee YE, Yates CJ (2011). A statistical explanation of MaxEnt for ecologists. Divers Distrib.

[47] Wu Y-M, Shen X-L, Tong L, Lei F-W, Mu X-Y, Zhang Z-X (2021). Impact of past and future climate change on the potential distribution of an endangered montane shrub Lonicera Oblata and its conservation implications. Forests.

[48] Phillips SJ, Anderson RP, Schapire RE (2006). Maximum entropy modeling of species geographic distributions. Ecol Model.

[49] Hijmans RJ, Phillips S, Leathwick J, Elith J, Hijmans MRJ. Package ‘dismo,’ *Circles*, vol. 9, no. 1, pp. 1–68, 2017.

[50] Team RC. R: A Language and Environment for Statistical Computing, Vienna, Austria, http://www.R-project.org/. 2018.

[51] Makki T et al. Impacts of climate change on the distribution of riverine endemic fish species in Iran, a biodiversity hotspot region. Freshw Biol, 2023.

[52] Maruthadurai R, Das B, Ramesh R (2023). Predicting the invasion risk of rugose spiraling whitefly, Aleurodicus Rugioperculatus, in India based on CMIP6 projections by MaxEnt. Pest Manag Sci.

[53] Valavi R, Elith J, Lahoz-Monfort JJ, Guillera-Arroita G. blockCV: An r package for generating spatially or environmentally separated folds for k-fold cross-validation of species distribution models, *Methods in Ecology and Evolution*, vol. 10, no. 2, pp. 225–232, 2019, 10.1111/2041-210X.13107.

[54] Makki T, Mostafavi H, Matkan A, Aghighi H (2021). Modelling climate-change impact on the spatial distribution of Garra rufa (Heckel, 1843)(Teleostei: Cyprinidae). Iran J Sci Technol Trans A: Sci.

[55] Liu C, White M, Newell G (2013). Selecting thresholds for the prediction of species occurrence with presence-only data. J Biogeogr.

[56] Valavi R, Guillera-Arroita G, Lahoz-Monfort JJ, Elith J. Predictive performance of presence-only species distribution models: a benchmark study with reproducible code. Ecol Monogr. 2022;92(1). 10.1002/ecm.1486.

[57] Lobo JM, Jiménez-Valverde A, Real R (2008). AUC: a misleading measure of the performance of predictive distribution models. Glob Ecol Biogeogr.

[58] jun Yi Y, Cheng X, Yang ZF, Zhang SH (2016). Maxent modeling for predicting the potential distribution of endangered medicinal plant (H. riparia Lour) in Yunnan, China. Ecol Eng.

[59] Fois M, Cuena-Lombraña A, Fenu G, Bacchetta G (2018). Using species distribution models at local scale to guide the search of poorly known species: review, methodological issues and future directions. Ecol Model.

[60] Phillips SJ (2009). Sample selection bias and presence-only distribution models: implications for background and pseudo-absence data. Ecol Appl.

[61] Pearson RG, Raxworthy CJ, Nakamura M, Townsend Peterson A (2007). Predicting species distributions from small numbers of occurrence records: a test case using cryptic geckos in Madagascar. J Biogeogr.

[62] Abdelaal M, Fois M, Fenu G, Bacchetta G (2019). Using MaxEnt modeling to predict the potential distribution of the endemic plant Rosa Arabica Crép. In Egypt. Ecol Inf.

[63] Phillips SJ, Dudík M (2008). Modeling of species distributions with Maxent: new extensions and a comprehensive evaluation. Ecography.

[64] Mirinejad S, Jahantab E, Mahmoudi MR, Navaei MN, Rahimi MM, Sharafatmandrad M (2018). Investigating the impact of some habitat characteristics on distribution of Stachys pilifera benth using the BMLR model in Iran. Pol J Environ Stud.

[65] Yan X, Wang S, Duan Y, Han J, Huang D, Zhou J (2021). Current and future distribution of the deciduous shrub Hydrangea macrophylla in China estimated by MaxEnt. Ecol Evol.

[66] Esfanjani J, Ghorbani A, Moameri M, Zare Chahouki MA, Esmali Ouri A, Ghasemi ZS (2021). Application of modeling techniques for the identification the relationship between environmental factors and plant species in rangelands of Iran. Ecol Inf.

[67] Abdi E, Saleh HR, Majnonian B, Deljouei A (2019). Soil fixation and erosion control by Haloxylon Persicum roots in arid lands, Iran. J Arid Land.

[68] Deljouei A, Cislaghi A, Abdi E, Borz SA, Majnounian B, Hales TC (2023). Implications of hornbeam and beech root systems on slope stability: from field and laboratory measurements to modelling methods. Plant Soil.

[69] Farahnak M (2019). Soil hydraulic conductivity differences between upslope and downslope of two coniferous trees on a hillslope. J for Res.

[70] Oke OA, Thompson KA (2015). Distribution models for mountain plant species: the value of elevation. Ecol Model.

[71] Santos X (2006). Inferring habitat-suitability areas with ecological modelling techniques and GIS: a contribution to assess the conservation status of Vipera latastei. Biol Conserv.

[72] Douaihy CB, Restoux G, Machon N, Dagher-Kharrat MB (2013). Ecological characterization of the Juniperus excelsa stands in Lebanon. Ecologia Mediterranea.

[73] Zeng XH, Zhang WJ, Song YG, Shen HT (2014). Slope aspect and slope position have effects on plant diversity and spatial distribution in the hilly region of Mount Taihang, North China. J Food Agric Environ.

[74] Najafzadeh R, Rashidi Z, Shokri B, Abdi H (2020). Investigation of morphological and ecological and essential oil content variation of some populations of thyme species (Thymus spp.) in the northwest and west of Iran. Iran J Rangelands Forests Plant Breed Genetic Res.

[75] Yousefzadeh S, Abedi R, Mokhtassi-Bidgoli A (2021). Which environmental factors are more important for geographic distributions of Thymus species and their physio-morphological and phytochemical variations?. Arab J Geosci.

[76] Corticchiato M, Tomi F, Bernardini AF, Casanova J (1998). Composition and infraspecific variability of essential oil from Thymus herba barona Lois. Biochem Syst Ecol.

[77] Boira H, Blanquer A (1998). Environmental factors affecting chemical variability of essential oils in Thymus piperella L. Biochem Syst Ecol.

[78] Arvin AA, Khodagholi M, Moazeni S (2020). Investigation of the bio-climatic needs of Thymus daenensis celak: the case of Isfahan Province. J Range Watershed Managment.

[79] Pirbalouti AG, Karimi A, Yousefi M, Enteshari S, Golparvar AR (2011). Diversity of Thymus daenensis Celak in central and west of Iran. J Med Plants Res.

[80] Tolyat MA, Tavakkol Afshari R, Jahansoz MR, Nadjafi F, Naghdibadi HA (2014). Determination of cardinal germination temperatures of two ecotypes of Thymus daenensis subsp. daenensis. Seed Sci Technol.

[81] Soilhi Z, Sayari N, Benalouache N, Mekki M (2022). Predicting current and future distributions of Mentha pulegium L. in Tunisia under climate change conditions, using the MaxEnt model. Ecol Inf.

[82] Gao X, Liu J, Huang Z (2022). The impact of climate change on the distribution of rare and endangered tree Firmiana kwangsiensis using the Maxent modeling. Ecol Evol.

[83] Bin Dong P (2022). Distributional response of the Rare and Endangered Tree species Abies chensiensis to Climate Change in East Asia. Biology.

[84] Tohidi B, Rahimmalek M, Arzani A (2017). Essential oil composition, total phenolic, flavonoid contents, and antioxidant activity of Thymus species collected from different regions of Iran. Food Chem.

[85] Tohidi B, Rahimmalek M, Trindade H (2019). Review on essential oil, extracts composition, molecular and phytochemical properties of Thymus species in Iran. Ind Crops Prod.

[86] Nadjafi F, Tabrizi L, Shabahang J, Damghani AMM. Cardinal germination temperatures of some medicinal plant species. Seed Technol, pp. 156–63, 2009.

[87] Kong F, Tang L, He H, Yang F, Tao J, Wang W (2021). Assessing the impact of climate change on the distribution of Osmanthus fragrans using Maxent. Environ Sci Pollut Res.

[88] Khwarahm NR (2020). Mapping current and potential future distributions of the oak tree (Quercus aegilops) in the Kurdistan Region, Iraq. Ecol Processes.

[89] Hamid M, Khuroo AA, Charles B, Ahmad R, Singh CP, Aravind NA (2019). Impact of climate change on the distribution range and niche dynamics of himalayan birch, a typical treeline species in Himalayas. Biodivers Conserv.

[90] Naghipour Borj AA, Ostovar Z, Asadi E (2019). The influence of climate change on distribution of an endangered medicinal plant (Fritillaria Imperialis L.) in central Zagros. J Rangel Sci.

[91] Baskin CC, Baskin JM. Seeds: ecology, biogeography, and, evolution of dormancy and germination. Elsevier; 1998.

[92] Shim SI, Moon J-C, Jang CS, Raymer P, Kim W (2008). Effect of potassium nitrate priming on seed germination of seashore paspalum. HortScience.

[93] Koutecká E, Lepš J (2009). Effect of light and moisture conditions and seed age on germination of three closely related Myosotis species. Folia Geobotanica.

[94] Phartyal SS, Thapliyal RC, Nayal JS, Joshi G (2003). Seed storage physiology of himalayan elm (Ulmus Wallichiana): an endangered tree species of tropical highlands. Seed Sci Technol.

[95] Parmesan C, Yohe G. A globally coherent fingerprint of climate change impacts across natural systems, *nature*, vol. 421, no. 6918, pp. 37–42, 2003. 10.1038/nature01286 12511946

[96] Thomas CD (2004). Extinction risk from climate change. Nature.

[97] Muñoz AR, Márquez AL, Real R (2013). Updating known distribution models for forecasting Climate Change Impact on Endangered species. PLoS ONE.

[98] Sharifi Ashoorabadi E, Mackizadeh Tafti M, Hasani J, Lebaschy MH (2021). Effect of temperature and humidity on seed germination of six different Thymus species. Iran J Seed Sci Technol.

[99] Abolmaali SM-R, Tarkesh M, Bashari H (2018). MaxEnt modeling for predicting suitable habitats and identifying the effects of climate change on a threatened species, Daphne mucronata, in central Iran. Ecol Inf.

[100] Khanum R, Mumtaz AS, Kumar S (2013). Predicting impacts of climate change on medicinal asclepiads of Pakistan using Maxent modeling. Acta Oecol.

[101] Fois M, Cuena-Lombraña A, Fenu G, Cogoni D, Bacchetta G (2018). Does a correlation exist between environmental suitability models and plant population parameters? An experimental approach to measure the influence of disturbances and environmental changes. Ecol Ind.

[102] Zhao Q, Li R, Gao Y, Yao Q, Guo X, Wang W (2018). Modeling impacts of climate change on the geographic distribution of medicinal plant Fritillaria Cirrhosa D. Don. Plant Biosystems-An Int J Dealing all Aspects Plant Biology.

[103] Sanjerehei MM, Rundel PW (2017). The impact of climate change on habitat suitability for Artemisia sieberi and Artemisia aucheri (Asteraceae)—A modeling approach. Pol J Ecol.

[104] Koch R, Almeida-Cortez JS, Kleinschmit B (2017). Revealing areas of high nature conservation importance in a seasonally dry tropical forest in Brazil: combination of modelled plant diversity hot spots and threat patterns. J Nat Conserv.

[105] Baldwin RA (2009). Use of maximum entropy modeling in wildlife research. Entropy.

[106] Qin A (2017). Maxent modeling for predicting impacts of climate change on the potential distribution of Thuja Sutchuenensis Franch., an extremely endangered conifer from southwestern China. Global Ecol Conserv.

[107] Tucker MA (2018). Moving in the Anthropocene: global reductions in terrestrial mammalian movements. Science.

[108] Li GQ, Liu CC, Liu YG, Yang J, Zhang XS, Guo K (2013). Advances in theoretical issues of species distribution models. Acta Ecol Sin.

[109] Lissovsky AA, Dudov SV (2021). Species-distribution modeling: advantages and limitations of its application. 2. MaxEnt. Biology Bull Reviews.

